# The Association Between Glycated Hemoglobin (HbA1c) Level and Vitamin D Level in Diabetes Mellitus Patients: A Cross-Sectional Study

**DOI:** 10.7759/cureus.47166

**Published:** 2023-10-17

**Authors:** Reem Mohammed Alqahtani, Ebtehag Faham Alsulami

**Affiliations:** 1 Department of Family Medicine, Faculty of Medicine, King Abdulaziz University, Jeddah, SAU; 2 Department of Family Medicine, King Abdulaziz University Hospital, Jeddah, SAU

**Keywords:** cross-sectional, vitamin d, saudi arabia, diabetes mellitus, deficiency

## Abstract

Background: Prior research has established noteworthy correlations between inadequate glycemic management and a multitude of problems in individuals diagnosed with diabetes mellitus (DM).

Methods: This is a cross-sectional retrospective study that was conducted at the Jeddah Center for the Care of Diabetes and Blood Pressure Patients, Jeddah, Kingdom of Saudi Arabia. The medical records of patients diagnosed with DM between 2015 and 2022 were identified and reviewed for the purpose of this study. Pearson correlation coefficient was used to examine the correlation between glycated haemoglobin (HbA1c) and vitamin D levels. Multiple linear regression analysis was applied to identify the association between HbA1c and vitamin D levels.

Results: A total of 152 patients were included in this study. The mean HbA1c level for the patients in this study was 8.2% (SD: 1.7). The median vitamin D level for the patients was 20.9 ng/ml (interquartile range (IQR): 13-30.4). More than half of the patients (n= 92; 60.5%) were found to have vitamin D insufficiency. Pearson correlation coefficient identified that there is an inverse correlation between the level of HbA1c and vitamin D level (r= -0.21 (95%CI -0.36 to -0.06; p-value= 0.007). Multiple linear regression analysis (adjusting for age and type of DM) identified that poor glycaemic control has a negative association with vitamin D level (regression coefficient (B) = -0.027; 95%CI -0.053 to - 0.001; p-value= 0.039).

Conclusion: Poor glycaemic control is associated with vitamin D deficiency in DM patients. It is recommended that patients with DM adhere to their medications and maintain a healthy lifestyle in order to manage their condition. This will improve their overall health, specifically their vitamin D status.

## Introduction

Diabetes mellitus (DM) is a group of metabolic disorders distinguished by chronic high blood glucose levels (hyperglycemia) resulting from the impairment of insulin action, insulin secretion, or both. This disruption leads to abnormalities in proteins, lipids, and carbohydrates metabolism [1,2]. As the most widespread metabolic disease globally, DM affects approximately 537 million adults worldwide, with projections indicating an increase to 783 million by 2045 [3]. The significant prevalence of DM has led to a considerable global impact on society and the economy. Addressing the prevention and treatment of DM has emerged as a foremost priority within the public health of numerous nations.

In tandem with the diabetes epidemic, vitamin D insufficiency has become a pervasive issue on a global scale. Recent years have seen an increase in attention to vitamin D. According to estimates, approximately one billion individuals globally suffer from either vitamin D insufficiency or deficiency [4]. Typically, vitamin D deficiency is characterized by having a serum 25 hydroxyvitamin D (25(OH) D) level below 20 ng/mL (50 nmol/L), and moderate deficiency is indicated by 25(OH) D levels below 10 ng/mL (25 nmol/L) [4]. This deficiency can exacerbate various health conditions and may even contribute to the development of diabetes [5,6]. Research has demonstrated a correlation between lower levels of 25(OH) D and an increased incidence of both DM [6] and metabolic syndrome [7]. Insulin sensitivity, secretion, and production are all impacted by vitamin D [8]. Moreover, several studies have noted a considerable decrease in the serum level of 25(OH) D among individuals with diabetes compared to those who are healthy [9-11].

DM is recognized as a significant public health concern in the Kingdom of Saudi Arabia (KSA), increasing in conjunction with the global diabetes pandemic [12,13]. According to the World Health Organization (WHO), KSA has the second-highest incidence rate of DM in the Middle East and ranks seventh globally. Within KSA, it is estimated that three million individuals or more have prediabetes, while the number of people with DM is estimated to be seven million. If left unaddressed, there is potential for DM prevalence to reach 50% within the Saudi population [14]. Additionally, vitamin D deficiency is too common among all population groups within KSA [15]. Earlier research has established a strong inverse correlation between serum vitamin D levels and glycemic control indicators, including fasting blood glucose (FBG) and glycated haemoglobin (HbA1c) levels [16-18]. Understanding the relationship between vitamin D levels and HbA1c in DM patients holds promise for enhanced therapeutic strategies and improved patient outcomes. Consequently, the principal objective of this study is to investigate the association between HbA1c levels and vitamin D levels in patients with DM in KSA.

## Materials and methods

Study design

This is a cross-sectional retrospective study that was conducted at the Jeddah Center for the Care of Diabetes and Blood Pressure Patients, Jeddah, KSA. The medical records of patients diagnosed with DM between 2015 and 2022 were identified and reviewed for the purpose of this study. Ethical approval for this study was obtained from the Institutional Review Board at the Saudi Ministry of Health in Jeddah, KSA (approval number: H-02-J-002).

Study population and settings

Male and female patients with type 1 and type 2 DM, aged 18 years and above, referred to the Jeddah Center for the Care of Diabetes and Blood Pressure Patients were included in this study, irrespective of the type of treatment. Patients with mental disorders or who did not give consent to participate in the study were excluded.

The Jeddah Center for the Care of Diabetes and Blood Pressure Patients was established in 2013. It provides tailored programs that are contingent upon the specific requirements of each patient. The care facility demonstrates adaptability by offering exercise programs of varying durations, ranging from one month to year-round, recognizing the unique needs of each patient. The organization focuses on providing specialized treatment for DM patients, both adults and children. The medical facility boasts a highly skilled team of healthcare professionals, including consultants, diabetic educators, and dieticians, who provide exceptional care to both adult and pediatric patients. The Center is dedicated to the prevention of diabetes among individuals at risk, as well as the provision of treatment for those who have already been diagnosed with the condition. The healthcare professionals working in it provide patients with the required knowledge and direction to effectively empower them in confronting the disease.

Outcomes

Patients' data were extracted from the medical records of the healthcare centre. Extracted data included age, gender, type of DM, comorbidities, and laboratory findings (HbA1c, vitamin D, cholesterol, triglycerides, and low-density lipoprotein (LDL) levels). Vitamin D deficiency was defined as a vitamin D level of less than 10 ng/ml, insufficiency was defined as a vitamin D level of 10-30 ng/ml, and sufficiency was defined as a vitamin D level of 30-100 ng/ml. HbA1c should be less than 7% (53 mmol/L) for non-pregnant people, according to the American Diabetes Association.

Statistical analysis

The normality of the data was checked using histogram, skewness, and kurtosis measures. Normally distributed continuous variables were presented as mean and SD. Not-normally distributed continuous variables were presented as median and interquartile range (IQR). The application of a log transformation was utilized in order to restore normality to skewed data for vitamin D levels. Pearson correlation coefficient was used to examine the correlation between HbA1c level and vitamin D level. Multiple linear regression analysis was applied to identify the association between HbA1c level and vitamin D level. A confidence interval (CI) of 95% (P<0.05) was applied to represent the statistical significance of the results, and the level of significance was predetermined as 5%.

## Results

Patients’ baseline characteristics

Table 1 presents patients’ baseline characteristics. A total of 152 patients were involved in this study. Almost half of them (n= 77; 50.7%) were males. The vast majority of the patients (n= 134; 88.2%) are diagnosed with type 2 DM. The most common comorbidity among the patients was hypertension, which accounted for 61.8% (n= 94).

**Table 1:** Patients’ baseline characteristics SD: Standard deviation

Variable	Frequency	Percentage
Gender (Males)	77	50.7%
Age (years), mean (SD)	55.8 (14.7) years	
Type of diabetes		
Type 1 diabetes mellitus	18	11.8%
Type 2 diabetes mellitus	134	88.2%
Comorbidities		
Hypertension	94	61.8%
Dyslipidaemia	87	57.2%
Cardiovascular diseases	5	3.3%
Kidney diseases	2	1.3%
Thyroid diseases	13	8.6%
Gastroesophageal reflux disease	3	2.0%

The mean HbA1c level for the patients in this study was 8.2% (SD: 1.7%). The median vitamin D level for the patients was 20.9 ng/ml (IQR: 13.0-30.4). For further details on the baseline characteristics of the patients, refer to Table 2.

**Table 2:** Laboratory findings SD: Standard deviation; IQR: Interquartile range; HbA1c: Glycated haemoglobin; LDL: Low-density lipoprotein

	Mean/Median	SD/IQR
Mean HbA1c level (%)	8.2%	1.7
Median vitamin D level (ng/ml)	20.9	13.0-30.4
Mean LDL (mg/dL)	102.1	29.5
Median cholesterol level (mg/dL)	179.5	1450-193.8
Median triglycerides level (mg/dL)	134.5	94.3-163.8

HbA1c and vitamin D status among the patients

Around 21.9% (n= 33) of the patients had an HbA1c below 7.0%. Almost one-third (n= 45) of the patients had an HbA1c level above 9.0%. More than half of the patients (n= 92; 60.5%) were found to have vitamin D insufficiency and 11.8% (n= 18) of them had vitamin D deficiency. Only 27.6% (n= 42) of the patients were vitamin D sufficient. Table 3 presents HbA1c and vitamin D levels among the patients.

**Table 3:** HbA1c and vitamin D status among the patients HbA1c: Glycated haemoglobin

Variable	Frequency	Percentage
HbA1c level categories		
Less than 7.0%	33	21.9%
7.0-7.4%	22	14.6%
7.5-8.0%	21	13.9%
8.1-8.4%	15	9.9%
8.5-9.0%	16	10.6%
Above 9.0%	45	29.8%
Vitamin D status		
Deficiency (<10 ng/ml)	18	11.8%
Insufficiency (10-30 ng/ml)	92	60.5%
Sufficiency (30-100 ng/ml)	42	27.6%

The correlation of HbA1c and vitamin D levels

Pearson correlation coefficient identified that there is an inverse correlation between the level of HbA1c and vitamin D level (r= -0.21; 95%CI -0.36 to -0.06; p-value= 0.007). Multiple linear regression analysis (adjusting for age and type of DM) identified that HbA1c level has a negative association with vitamin D level (regression coefficient (B)= -0.027; 95%CI -0.053 to - 0.001; p-value= 0.039).

## Discussion

This cross-sectional study revealed several significant findings regarding HbA1c and vitamin D status and the correlation between HbA1c levels and vitamin D levels in patients with DMs. Our study showed that approximately 21.9% of the studied patients exhibited HbA1c levels below 7%, indicating good glycemic control in this subgroup. On the other hand, almost one-third of the patients (approximately 30.3%) exhibited significantly elevated HbA1c levels above 9.0%, suggesting poor glycemic control in these individuals. Notably, a previous study identified significant associations between suboptimal glycemic control and many factors, including comorbidities, lack of blood glucose self-monitoring, inadequate physical activity, total cholesterol exceeding 200 mg/dl, antidiabetic medication types, diabetes duration of ≥7 years, and elevated waist to hip ratio [19]. Our results were in line with numerous earlier research, reported poor glycemic control among patients with type 1 DM according to HbA1c levels [20-24]. Also, according to the results of a prior study conducted in Saudi Arabia, glycemic control was poor in about 65% of patients with type 2 DM [25]. A previous study in Riyadh found that 67.7% of patients with type 2 DMs exhibited poor glycemic control [26]. Patients in Jazan and Al Hasa were found to have comparable percentages of 74% and 67.9%, respectively [27,28]. Other Middle Eastern settings such as Kuwait, the United Arab Emirates, Jordan, and Oman have also shown a high prevalence of poorly controlled diabetes, with rates of 78.8%, 69%, 65.1%, and 65%, respectively [29-32]. This collective evidence underscores the critical situation of poor glycemic control in Saudi Arabia and the broader Middle East, warranting attention due to its adverse health implications. However, earlier investigations in Saudi Arabia [33], Australia [34], and Brazil [23] revealed a positive link between participation in diabetes education programs and improved glycemic control.

The prevalence of vitamin D insufficiency and deficiency among the study participants is another significant aspect. In the current study, a substantial proportion of patients (60.5%) were found to have vitamin D insufficiency, while 11.8% of the patients exhibited vitamin D deficiency. In contrast, only 27.6% of the patients were identified as having sufficient vitamin D levels. These findings highlight the substantial prevalence of vitamin D insufficiency among patients with DM. Furthermore, these findings align with previous research indicating lower vitamin D levels in individuals with DM [35,36]. Previous studies found that among people with type 2 DM, the prevalence of vitamin D deficiency ranged from 63.5% to 91.1% [37,38].

According to previous studies, vitamin D deficiency is significantly common in otherwise healthy Saudi individuals, principally due to the following factors: advancing age, sedentary lifestyles, inadequate dietary vitamin D supplementation, obesity, lack of education, and inadequate sun exposure. These factors, in turn, affect bone turnover markers and bone mineral density [39,40]. Recent research has revealed that even young and middle-aged Saudi Arabian males could face severe health outcomes due to vitamin D deficiency. That is significant as a substantial prevalence of vitamin D deficiency persists among Saudi Arabians regardless of reporting adequate dairy product consumption and sufficient sunlight exposure [41]. Besides, the development of type 2 DM has more recently been linked to vitamin D deficiency, which has been linked to increased insulin resistance and reduced insulin secretion [42].

Our study revealed a statistically significant inverse correlation between HbA1c and vitamin D levels (r = -0.21, 95%CI -0.36 to -0.06, p-value = 0.007). This correlation suggests that as HbA1c levels increase, there is a tendency for vitamin D levels to decrease. These results are consistent with previous studies. An Italian research investigation revealed an inverse correlation between HbA1c and serum vitamin D levels among individuals with type 2 DM (r = -0.116, p = 0.003) [18]. Similarly, research conducted in the Gaza Strip demonstrated significant negative associations between serum vitamin D and HbA1c levels (r = -0.258, p = 0.015) [43]. However, this contrasts with earlier research that found no association between vitamin D and HbA1c [44].

To further explore the association between these variables, we conducted multiple linear regression analysis, adjusting for age and type of DM. The results of this analysis provided additional insight into the association. After accounting for potential confounders, we found that HbA1c levels maintained a negative association with vitamin D levels (B = -0.027, 95%CI: -0.053 to -0.001, p-value = 0.039); this indicates that the increase in HbA1c is negatively associated with a decrease in vitamin D levels. These findings suggest that the association between levels of HbA1c and vitamin D holds even when considering other influential factors. These findings are consistent with the results of multiple linear regression analyses from previous studies, which have reported a significant inverse association between HbA1c and vitamin D levels in Jordan [45] and Saudi Arabia [46].

Furthermore, a previous meta-analysis has indicated that supplementing with vitamin D could provide advantages in lowering homeostasis model assessment insulin resistance (HOMA-IR), HbA1c, and FPG among individuals with both type 2 DM and inadequate levels of vitamin D [47]. The enhancement of glycemic regulation attributed to vitamin D arises from its ability to mitigate peripheral insulin resistance, modify immune responses and systemic inflammation, and stimulate insulin secretion [48-50]. Considering the widespread prevalence of both DM and vitamin D deficiency in Saudi Arabia, healthcare providers should prioritize evaluating the vitamin D status of patients with DM, especially those facing challenges in achieving optimal glycemic control. Addressing vitamin D deficiency through supplementation could potentially provide a pathway for enhancing glycemic outcomes within this population.

This study has limitations. This is a single-center study. This might affect the generalizability of our study findings. The cross-sectional study design restricted our ability to investigate causality between study variables. Therefore, our findings should be interpreted carefully.

## Conclusions

There exists a negative correlation between inadequate glycemic management and vitamin D insufficiency in individuals diagnosed with DM. Individuals diagnosed with DM are recommended to consistently comply with their prescribed prescriptions and adopt a health-conscious lifestyle in order to effectively manage their condition. This intervention is expected to improve individuals' overall health outcomes, with a specific focus on enhancing their vitamin D levels.
